# Analysis of copy loss and gain variations in Holstein cattle autosomes using BeadChip SNPs

**DOI:** 10.1186/1471-2164-11-673

**Published:** 2010-11-29

**Authors:** Eyal Seroussi, Giora Glick, Andrey Shirak, Emanuel Yakobson, Joel I Weller, Ephraim Ezra, Yoel Zeron

**Affiliations:** 1 Institute of Animal Sciences, ARO, The Volcani Center, Bet Dagan 50250, Israel; 2 Israel Cattle Breeders Association, Caesaria Industrial Park 38900, Israel; 3 Sion, AI Institute, Shikmim 79800, Israel

## Abstract

**Background:**

Copy number variation (CNV) has been recently identified in human and other mammalian genomes, and there is a growing awareness of CNV's potential as a major source for heritable variation in complex traits. Genomic selection is a newly developed tool based on the estimation of breeding values for quantitative traits through the use of genome-wide genotyping of SNPs. Over 30,000 Holstein bulls have been genotyped with the Illumina BovineSNP50 BeadChip, which includes 54,001 SNPs (~SNP/50,000 bp), some of which fall within CNV regions.

**Results:**

We used the BeadChip data obtained for 912 Israeli bulls to investigate the effects of CNV on SNP calls. For each of the SNPs, we estimated the frequencies of occurrence of loss of heterozygosity (LOH) and of gain, based either on deviation from the expected Hardy-Weinberg equilibrium (HWE) or on signal intensity (SI) using the *PennCNV *"detect" option. Correlations between LOH/CNV frequencies predicted by the two methods were low (up to r = 0.08). Nevertheless, 418 locations displayed significantly high frequencies by both methods. Efficiency of designating large genomic clusters of olfactory receptors as CNVs was 29%. Frequency values for copy loss were distinguishable in non-autosomal regions, indicating misplacement of a region in the current BTA7 map. Analysis of BTA18 placed major quantitative trait loci affecting net merit in the US Holstein population in regions rich in segmental duplications and CNVs. Enrichment of transporters in CNV loci suggested their potential effect on milk-production traits.

**Conclusions:**

Expansion of HWE and *PennCNV *analyses allowed estimating LOH/CNV frequencies, and combining the two methods yielded more sensitive detection of inherited CNVs and better estimation of their possible effects on cattle genetics. Although this approach was more effective than methodologies previously applied in cattle, it has severe limitations. Thus the number of CNVs reported here for the Holstein breed may represent as little as one-tenth of inherited common structural variation.

## Background

The Holstein-Friesian breed is the world's highest-producing dairy cattle; much of its outstanding milk production was gained by selection of elite artificial insemination (AI) bulls based on breeding values that were estimated by progeny testing. Genomic selection is a newly developed tool for the estimation of breeding values through the use of genome-wide genotyping of single nucleotide polymorphisms (SNPs). Over 30,000 Holstein bulls have been genotyped with the Illumina BovineSNP50 BeadChip [1], which includes 54,001 SNPs (~SNP/50,000 bp). This chip may capture any genetic variance that is genetically linked to these markers, as well as copy number variations (CNVs) [2,3]. A CNV is a structural variation, including deletion, duplication, translocation or inversion. CNV has been recently identified in human and other mammalian genomes, and it is now recognized that CNV might be a major source of heritable variation in complex traits [4]. In humans, over 14,478 CNV loci have been recorded based on 89,427 different entries that cover about one-third of the genome. Of these entries, 65% include CNVs that range mostly between 1 and 10 kb and 34% are indels in the range of 100 bp to 1 kb http://projects.tcag.ca/variation/. CNV regions (CNVRs) encompassing adjacent or overlapping losses or gains cover 12% of the human genome. Hence, this source of variation has more nucleotide content per genome than SNPs [4]. However, assuming an average spontaneous CNV mutation rate of 1/10,000 per locus [5], it is expected that a considerable portion of the reported entries arise from *de novo *CNVs of a sporadic nature.

Several algorithms for CNV identification from SNP arrays are available [6]. Following reports that *PennCNV *was the most reliable algorithm in the detection of CNVs from Illumina BeadChip data [7,8], we chose this software to analyze signal intensity (SI) data. *PennCNV *is a CNV detection tool that incorporates multiple sources of information, including the ratio of total SI to allelic intensity at each SNP marker. This software was originally developed for Illumina whole-genome BeadChip arrays [9].

The introduction of AI to modern dairy herd management has resulted in a loss of genetic diversity in Holsteins and the effective size of the Holstein population is low (e.g. 39 in the USA [10]). Since the Israeli Holstein population has been under intensive selection for 50 years, its genetic pool is expected to have similar characteristics. Although it is now accepted that genomes vary more at the structural level than at the nucleotide-sequence level, little has been published on CNVs in Holsteins.

In a study that validated the quality of BovineSNP50 BeadChip performance [11], population-wide genotyping of Israeli Holstein bulls was initiated in order to introduce genomic selection into the Israeli breeding program. Our study makes use of these data to describe the frequent CNV in Holsteins and investigate its effect on BeadChip calls. We propose to combine Hardy-Weinberg equilibrium (HWE)-based and SI-based methods to reliably detect CNVs of the deletion and duplication types that are not *de novo *or sporadic CNVs, which are less likely to be of any economic value.

## Results and Discussion

### HWE-based detection of CNV

We used the data obtained for 912 Holstein bulls to investigate the effects of CNVs on BovineSNP50 BeadChip calls. For each of the SNPs, we estimated the frequency of occurrence of deletions and insertions using a generalization of the Hardy-Weinberg principle for more than two allele frequencies (p, q) by assuming presence of a third allele (r). Under the assumption of three-alleles, expected HWE frequencies are obtained by the trinomial expansion of (p + q+ r)^2 ^= 1. Defining 'n' as the number of individuals sampled, 'pq_o_' as the detected number of individuals with phenotype similar to the pq heterozygote phenotype divided by n, 'p_o_' as the number of allele p-like homozygotes divided by n, and 'q_o_' as the number of allele q-like homozygotes divided by n, the solution for this expansion in the case of a null allele r is: r_l _= [0.25 - 0.25pq_o _+ p_o_q_o_/pq_o_]^0.5 ^- 0.5; in the case of gain of an allele which consists of both types, r is: r_g _= [p_o _+ q_o _+ pq_o_]^0.5 ^- p_o_^0.5 ^- q_o_^0.5 ^(see Additional file 1 for a detailed mathematical solution and Additional file 2 for allele distribution, χ^2 ^test, and r_l _and r_g _values for all 54,001 SNPs of the Illumina BovineSNP50 BeadChip). Average values of r_l _and r_g _for autosomal markers were -0.3% ± 2.7 and 0.5% ± 2.1, respectively. For non-pseudoautosomal markers on the X chromosome (positions 28,044-86,115,497), where the model of inheritance does not fit the model under which the formulas were developed, average values of r_l _and r_g _were 361% ± 258 and -25% ± 16, respectively. Thus, encountering extreme values (above 100%) for r_l_, when analyzing autosomal markers, may indicate an error in the mapping of markers that are actually located on sex chromosomes. Although the HWE deviation is an important factor in CNV occurrence, other reasons than erroneous positioning of markers of sex chromosomes may also exist; for example, systematic problems in distinguishing the alleles, due to technical failures. However these are unlikely, because of the high quality of the Beadchip technology [11].

### SI-based detection of CNV

Using the *PennCNV *detection module, we analyzed the autosomes of each of the 912 bulls for CNVs. From the output of this analysis, which contained the chromosomal positions and copy numbers of the detected CNVs, the frequency of loss or gain for each SNP marker was calculated (Additional file 2). Average loss and gain values for autosomal markers were l_si _= 0.02% ± 0.2 and g_si _= 0.12% ± 0.15, respectively.

### Comparing the HWE-based and SI-based methods for CNV detection

**Figure 1:**
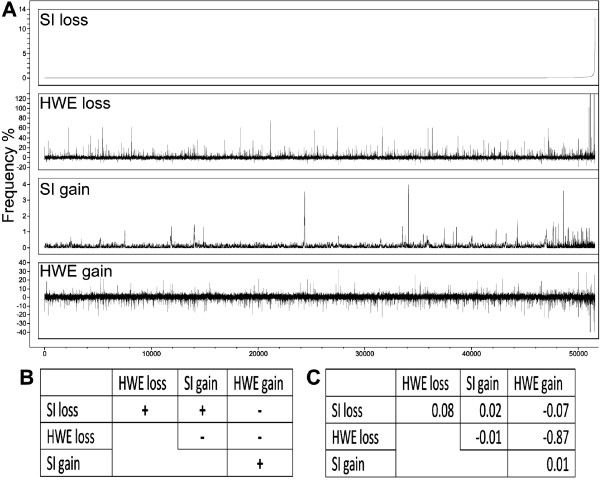
**Distribution and correlation of the LOH/CNV frequency values predicted by the HWE-based and SI-based methods**. (A) For 54,001 markers, the frequencies of copy loss (SI loss) and gain (SI gain) were calculated based on signal intensities using the output from *PennCNV *analysis. Frequencies of copy loss (HWE loss) and gain (HWE gain) were calculated based on a generalization of the Hardy-Weinberg principle for more than two allele frequencies by considering an extra allele frequency. These frequencies are presented as an overlay plot in which the X scale represents the 54,001 markers first sorted by the SI loss values and then by chromosomal position. (B) Table describing the expected signs for correlation between the methods for predicting CNV frequency. (C) Observed correlations between these methods.

Seeking confirmation of the CNV detection, we examined the correlation between the HWE-based and SI-based detection methods (Figure 1). When sorted according to frequency of deletion as detected by the *PennCNV *analysis (SI loss), the markers that exhibited frequent LOH using the HWE-based formulas (high r_l, _and negative r_g_) clustered together towards the right end of panel A (Figure 1). The distribution of frequencies of the SI-based method was of limited range (from 0 to 14%) compared to the HWE-based method (Figure 1A). Six autosomal markers displayed r_l _values higher than 100%, five of them that were closely mapped on BTA7 (see BTA7 section). These markers were regarded as non-autosomal and removed from further analyses.

As the Holstein population has a very low effective population size, it was expected that some of the CNV alleles within our sample would be common. However, all of the CNVs detected by *PennCNV *were relatively rare (Figure 1A),) and we suspected that this may arise from setting up input parameters that are too stringent. Therefore, we examined several setups for running this program using different HMM input files and different length restrictions on the CNV chromosomal size (data not shown). We then calculated the correlations for each setup, with no limit on CNV size. We adopted the setup that gave the highest correlations between the SI-based and HWE-based methods with the expected direction signs (Figure 1B). Since "negative loss" is equal to gain, it was generally expected that negative correlations would be displayed when comparing the loss and gain methods. SI gain and loss involves a simple count of events, and therefore could only yield positive correlations when CNV exhibited both LOH and duplication alleles. A negative correlation (r = -0.87) was indeed observed between the HWE-based gain and loss methods, indicating that the equations presented for calculation of loss and gain frequencies also function well for loci that do not fit the model for which they were developed. For example, in a locus where LOH was frequent, the absolute value of r_g _would be similar to r_l _but with a negative sign. Correlations between the LOH/CNV frequencies predicted by the HWE-based and SI-based methods was low (up to r = 0.08). Nevertheless, a number of loci displayed above-average CNV frequencies by both HWE-based and SI-based methods simultaneously. The discrepancy between the methods may be explained by random deviations from HWE (minor deviation from HWE could have also arisen of selection); by *de novo *CNVs that do not affect HWE; and by the conservative thresholds on the detection of CNVs by *PennCNV*, which presents moderate power with a low false-positive rate [6].

### Combining the results of HWE-based and SI-based methods

A total of 221 markers displaying frequencies that were more than one standard deviation (SD) above average for LOH (2.33% and 0.22% for r_l _and l_si_, respectively) were included in the data set for regions of LOH variation. A total of 515 markers displaying frequencies that were more than one SD above average for duplication (2.67% and 0.27% for r_g _and g_si_, respectively) were included in the data set for copy gains. Since these markers tended to cluster together, and CNVs may affect expression of genes that are up to 0.5 kb away [12], such adjacent markers were assigned to the same CNVR (Additional file 3). These two data sets were combined and compared to the available CNV annotations in cattle (Additional file 3, LOH and CNV sets are labelled in red and blue, respectively; yellow and white labels indicate previously published CNVs that were confirmed to be within 0.5 kb, or not detected in this study, respectively). The actual length of the predicted CNVs cannot be accurately assigned using the BeadChip data, and CNV of a region that is evident from a single marker may belong to a region shorter than 1000 bp, which is usually referred to as an indel. In total, we detected 169 indels/CNVs/CNVRs of copy losses (LOH) and 246 of copy gains. These were compared to 86 documented cattle CNVs with recorded frequency above 2.5% [2,3], and with 141 frequent CNVRs [13]. The latter study analyzed only 20 individuals, with an average frequency of detection of 3 ± 2: we assumed that CNVRs detected in three or more individuals are likely to be frequent. Defining confirmation as co-occurrence of a documented CNV within 0.5 kb of a CNV detected here, 32 (37%) of the CNVs reported in [2] and [3], and 28 (20%) of those reported in [13] were confirmed by the present study. Another line of evidence supporting our list of LOH variations was that most (68%) of these markers had significantly high rates of missing calls. The average for "no calls" was 15 ± 39 out of 912 bulls genotyped for the autosomal markers, while for the 221 selected LOH markers, the average was 201. An increase in no-call rate is expected with an increase in the frequency of null alleles, as individuals that are homozygous for the null allele should fall within the no-call category and the expected number of individuals with no call should be ≥nr_l_^2 ^(see Additional file 1). The higher than expected frequency of SNPs with deviation from the expected HWE frequencies calculated using the χ^2 ^test was also an indication of CNVs. There were 47,154 autosomal polymorphic SNPs, of which 4,486 (9.5%) had probabilities <0.05 for HWE. Despite selection against non-HWE SNPs during the BeadChip preparation [14], their fraction is nearly double that expected by chance. While overall, 9.5% of the autosomal polymorphic BeadChip markers had probabilities <0.05, frequencies for markers meeting this criterion in the lists of LOH (221) and CNV gain (515) were 83% and 51%, respectively.

### BTA7

**Figure 2:**
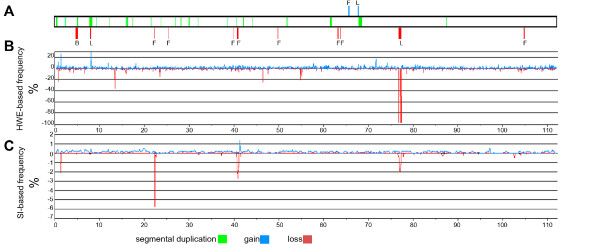
**CNV analysis of BTA7**. Delineations of BTA7 (X scale) were used to indicate copy loss and gain in red or blue, respectively. (A) Segmental duplications [2] were annotated in green. Common CNVs (frequency > 2.5%) previously detected using genomic and CGH analyses [2,3,13] were marked by letters "L", "B" and "F", respectively. (B) LOH and copy gain frequencies were calculated using the trinomial expansion of HWE and 912 BeadChip samples. For each marker, only positive values for copy loss (r_l_) and gain (r_g_) are presented. (C) Loss and gain frequencies (l_si _and G_si_) were also calculated based on the *PennCNV *analysis for these samples.

Along the autosomes, we encountered the highest values for r_l _on BTA7. We therefore compared the frequencies of CNV occurrence estimated by the HWE-based and SI-based methods and the previously described segmental duplications and common (frequency > 2.5%) CNVs (Figure 2). Previous data were based mostly on the sequence data of bovine genome assemblies (Btau_4.0 and UMD3), array comparative genomic hybridization (CGH) [2] and the BovineSNP50 BeadChip [3]. Low correlations were observed between the CNV-detection methods, except for the chromosome interval that included positions (76,944,037-77,340,598) containing genes similar to melanoma antigen (*MAGEB*). Virtually no heterozygotes were detected for the five polymorphic SNP markers within this interval. The HWE- calculated LOH frequencies for these exceeded 100%, values which are typical for non-autosomal chromosomes. Indeed the human *MAGEB *orthologs are mapped to chromosome X, suggesting that in current bovine genome assemblies, the X chromosomal region containing copy variation of this gene is misplaced and that the repetitive character of this locus may have complicated its chromosomal assignment.

### BTA18

**Figure 3:**
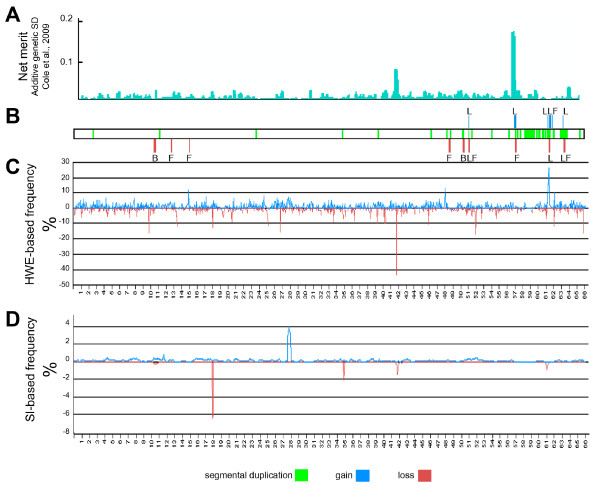
**Marker effect on net merit and CNV on BTA18**. (A) The largest effect of all the BeadChip SNPs on production traits important for net merit was previously mapped to BTA18 and marker effects for net merit within this chromosome were previously calculated [15]. Panels B, C, D and E were produced as described in the legend for Fig. 2A, B and C, respectively.

Marker effects indicated the importance of BTA18 for economic merit according to the USDA index [15]. The most pronounced effect was associated with a QTL related to calf size or birth weight in position 57,125,868 (Figure 3A). To exemplify the possible association of economic traits with CNVs, we also present detailed results for BTA18 (Figure 3). While the major peak (57,125,868) coincided with a region rich in segmental duplications within a CNV gain (frequency 54%, positions 57,092,062-57,270,472, [2], Figure 3B), the second effect peak (41,453,097, SD = 0.07) was mapped to a region where LOH was detected in this study by both methods, with maximal value at position 41,760,794: at this position, there was a HWE-calculated loss of 43% and a SI-calculated loss of 1% (Figure 3C and 3D). Hence, the proximity of these largest effects to CNVs suggests association of these copy gain and loss regions with traits of economic merit.

### Analysis of gene content within frequently detected CNVs

In our study, some of the detected CNVs may have arisen from spontaneous CNV mutations. High rate of *de novo *mutations for several human diseases caused by CNV has been observed [5]. *De novo *mutations may also explain the low rate of verification (<10%) of candidate CNVs detected by SNP arrays reported in human studies [16]. To reliably detect CNVs that are expected to have a functional impact and are not *de novo *or sporadic, we targeted those that are frequently detected by both HWE-based and SI-based methods. Nevertheless, the sporadic occurrence of CNVs in the genomes of ancestral key bulls in regions that are neutral for selection are expected to result in some frequent CNVs that have no function. Indeed, we could not associate any functional gene with 61 (15%) of the common CNVs that we reported. These are indicated in the corresponding gene column as 'none' or pseudogene (Additional file 3).

Other CNVs demonstrated an enrichment of functions which was associated with overrepresentation of specific gene families (Table 1, purple, yellow and green backgrounds). Gene content of CNVRs (Additional file 3) demonstrated an overrepresentation of genes/pseudogenes for olfactory receptors (ORs, 36 genes), cadherins (10 genes) and transporters (63 genes, mostly including solute carriers and ABC transporters). Enrichment for ORs and ABC transporters in CNVRs has been previously described for cattle CNVs [2]. Variation in ABC transporters may affect milk content [17]. The tendency of cadherins to accumulate CNVs may relate to their highly repetitive structure containing cadherin, laminin A and G, EGF and mucin repeats [18], which may be a source of genomic instability.

**Table 1:** Gene ontology (GO) categories significantly overrepresented in Holstein CNV.

	Human ref. gene #	CNV gene #	Expected	*P *value
***Pathways***				

Wnt signalling pathway^1^	348	26	10.80	0.00878

Alzheimer disease-presenilin pathway^1^	143	12	4.44	0.344

Cadherin signalling pathway^1^	168	13	5.21	0.459

***Biological Process***				

Chemosensory perception^2^	207	37	6.42	8.75E-15

Olfaction^2^	198	36	6.14	1.88E-14

Sensory perception^2^	506	48	15.70	7.16E-10

G-protein-mediated signalling^2^	834	66	25.87	2.10E-09

Cell surface receptor-mediated signal transduction^2^	1638	92	50.82	6.01E-06

Signal transduction^2^	3406	156	105.67	1.26E-05

Transport^3^	1306	63	40.52	0.014

***Molecular Function***				

G-protein-coupled receptor^2^	571	50	17.72	2.18E-08

Receptor^2^	1512	82	46.91	2.77E-05

Cell-adhesion molecule^1^	395	24	12.25	0.0511

Cadherin^1^	111	11	3.44	0.14

Transporter^3^	648	32	20.10	0.229

NCBI list of 25,431 *Homo sapiens *genes were compared to the list of 789 genes orthologous to the CNV gene content (Additional file 3) using PANTHER. ^1,2,3 ^denote categories that are associated with overrepresentation of gene families for cadherins, olfactory receptors and transporters, respectively. The full version of this table is provided in Additional file 4.

### Analyzing gene clusters of ORs as a measure of the effectiveness of common CNV discovery

**Figure 4:**
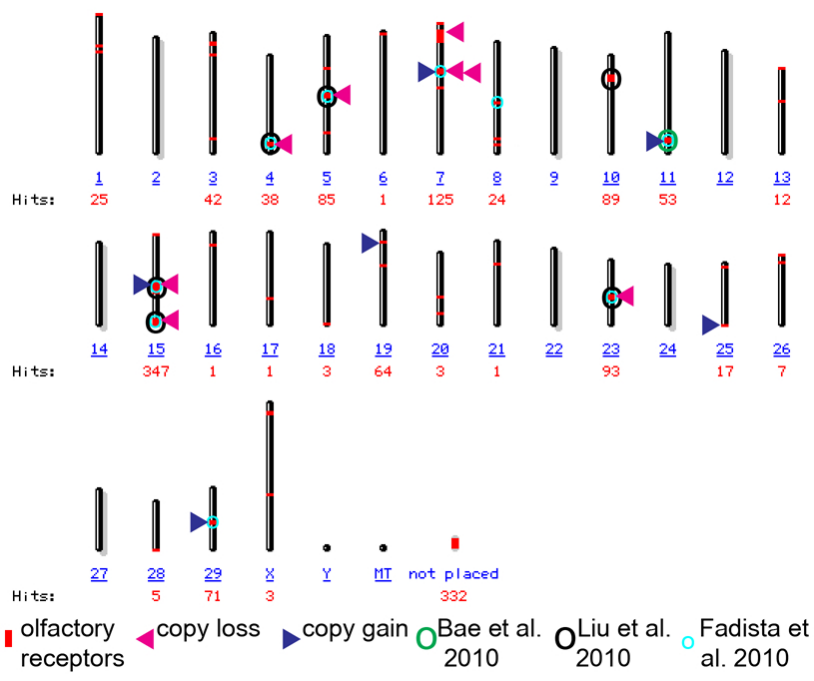
**CNVs within olfactory receptor (OR) clusters**. NCBI map viewer http://www.ncbi.nlm.nih.gov/projects/mapview/map_search.cgi?taxid=9913 was searched for the string "olfactory receptor" and the output was the base for the presented genome view of OR locations (red boxes). For each chromosome, the number of database hits (red font), including all types of redundant map elements, was indicated under the corresponding chromosome number (underlined blue font). Common OR copy losses and gains detected in this study are indicated with red and blue arrowheads, respectively. Circles around OR clusters indicate CNVRs (frequency > 2.5%) which have been previously observed in cattle autosomes.

The significantly pronounced (*p *< 2E-14) enrichment for olfaction was due to the frequent occurrence of CNVs in gene clusters for ORs [19]. Organization of OR gene clusters is well conserved among mammals and despite the difference in the number of genes, 34 large genomic clusters (≥5 ORs) are present in humans and mice [20]. Thus, the rate of assignment of CNVs into these clusters may indicate the effectiveness of CNV detection in general. Common OR CNVs that were detected in this study, as well as those that have been previously observed in cattle, were labelled on the map of ORs in the bovine genome, which contained 40 distinct autosomal locations (Figure 4). Assuming that the 34 large autosomal OR clusters are common CNVRs in all breeds, the efficiency of detection of this study was 29% while previous studies annotated 18%, 3% and 26% as frequent CNVRs [[2,3,13], respectively]. However, when considering CNVs of smaller size, microarray analysis at a resolution of ≤85,000 probes may detect fewer than 10% of all CNVs [5], and it is likely that when designing the Illumina BeadChip, probes in CNVRs that were not in HWE were selected against [14]. Therefore, the 418 CNVs in Holsteins reported here may be part of a 10-fold larger repertoire of inherited common CNVs, which have yet to be described. Moreover, our HWE-based method is only suitable for polymorphic sites, and not for copy gain variation in which the duplicated copy does not differ from the source copy. Another limitation of our HWE approach is that only two simplified models of one copy loss or gain were considered, while much more complex scenarios involving both loss and gain of multiple copies have been frequently observed [2]. Despite these rather serious limitations, the number of frequent CNVs detected in this study exceeds previous reports for the following possible reasons: 1) our bull sample was larger and belonged to a homogenous population; 2) the noisy nature of the data obtained from previous SI-based hybridization experiments called for a conservative interpretation; 3) the equations based on HWE for calculation of CNV frequency may yield true results in CNV loci that do not exactly fit the model (e.g. negative frequency was encountered when the equation for copy loss was used for a locus with a copy gain). Recent studies in humans indicated that copy number analysis using next-generation sequencing is more accurate than array-based platforms, in determination of absolute copy number and break-point structure [21]. With sequencing technology allowing more sequence reads at lower costs, it is likely to become the method of choice for CNV analysis, which would enable the uncovering of the full extent of inherited structural variation in cattle.

### Validation of copy number variation and of CNV association with breeding values using qPCR

**Figure 5:**
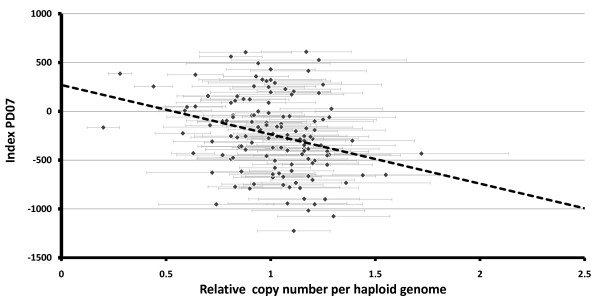
**Validation of CNVR#456 on BTA18 and its association with breeding values**. Relative copy number per haploid genome was calculated for 132 sires using real-time qPCR of amplicon I from CNVR#456 near SNP position 41,760,794 on BTA18. Dashed line denotes the linear regression of these sires' breeding values for the Israeli total merit index (PD07) on copy number of amplicon I (p < 8 × 10^-4^).

Further validation of the effectiveness of our approach of combining HWE-based and SI-based methods for CNV detection was obtained by real-time qPCR analysis of the region where LOH was detected by this study with maximal value at position 41,760,794 on BTA18. Relative copy numbers per haploid genome (CNRQs) were estimated for 160 sires randomly selected from the sample analysed in beadchip experiment. Two amplicons, about 150 Kbp apart, within the relevant CNVR #456 (additional file 3) were analysed. Results of the validation of CNV at amplicon I and of its association with breeding values for the Israeli index of total merit are presented in Figure 5. In addition to the significant association between the loss of this region and total merit (p < 0.0008), significant associations with copy number were also found with the genetic evaluations for protein production (p < 0.006), fat production (p < 0.001) and herd life (p < 0.007). These observations are in accordance with the QTL of Net merit observed for US Holsteins at this chromosomal position (Figure 3).

The CNRQs of two sires (3981, CNRQ = 0.28 ± 0.06; 7133, CNRQ = 0.2 ± 0.08) suggested that they were homozygous for the deletion. In the sample of 132 sires an estimate of frequency of the deletion allele can be derived as 12%, based on the occurrence of two homozygotes and assuming a Hardy-Weinberg distribution of genotypes. Since the number of homozygotes will have a Poisson distribution, the 95% confidence interval will not be symmetric, and extends from 0.6 to 7.2 homozygotes, which is equivalent to a confidence interval of 6.7% to 23.4% for the deletion allele. Thus, for this CNVR, the average LOH frequencies of the HWE-based and SI-based methods (Figure 3), was within the confidence interval.

Significant positive correlation (r = 0.5) was observed between CNRQs of the two amplicons in 100 sires that passed quality thresholds for both amplicons. This indicates that a portion of the sires analysed displayed CNV that spanned both amplicons, yet some of them may had other CNV alleles with borders that excluded one of the amplicons. Hence, the qPCR analysis of both amplicon supported the prediction of CNVR #456 in this work.

## Conclusions

Expansion of HWE and *PennCNV *analyses enabled an estimation of LOH/CNV frequencies, and combining these methods yielded better detection of inherited CNVs. Correlation between LOH/CNV frequencies predicted by the HWE-based and SI-based methods was low (up to r = 0.08). The highest correlation was observed for the minimal CNV length of 1 SNP for the *PennCNV *analysis. Under these conditions, 418 locations displayed significantly high frequency by both methods. Efficiency of designating large genomic clusters of ORs as CNVs was 29%. Frequency values for copy loss were distinguishable in non-autosomal regions and for the values obtained for BTA7 positions 76,944,037-77,340,598, suggesting misplacement of the X chromosomal region containing CNV of the melanoma antigen gene onto BTA7 in the current bovine genome assemblies. Analysis of BTA18 placed important net merit QTLs in regions rich in segmental duplications and CNVs. Enrichment of transporters in CNV loci suggested their potential effect on milk-production traits. Although our approach for identifying common CNVs was more effective than previous methodologies applied in cattle, it has severe limitations. Thus the number of CNVs reported here for the Holstein breed may be part of a 10-fold larger repertoire of inherited structural variation that has yet to be described.

## Methods

### BeadChip analysis

DNA was extracted from the semen of 912 Holstein bulls used for AI in Israel http://www.icba-israel.com/cgi-bin/bulls/en/bl_main.htm. These included sires born in Israel, as well as international sires originating from France (4), Germany (2), the Netherlands (26) and the USA (27). The sires' DNA was genotyped using BovineSNP50 BeadChip (Illumina, Inc.), which included 54,001 SNPs as described previously [11].

### HWE-based detection of common CNVs

Frequencies for copy loss (r_l_) and gain (r_g_) were calculated using the formulas r_l _= [0.25-0.25pq_o_+p_o_q_o_/pq_o_]^0.5 ^- 0.5 and r_g _= [p_o_+q_o_+pq_o_]^0.5 ^- p_o_^0.5 ^- q_o_^0.5 ^(see Additional file 1 for a detailed explanation). The r_l _value could also be calculated from the number of missing calls in cases in which no calls were observed as a result of a homozygous null allele, and not as a result of technical problems. However, the low correlation (r < 0.2) between r_l _values calculated by these two methods indicated that technical problems did in fact play a role in most of the observed missing calls. This prompted us to routinely use a sample size (n) computed as the number of sires that were successfully called according to the default settings of GenomeStudio. Using this n value in cases of frequent copy loss led to a slight increase in r_l _over the value obtained when using the total sample size (n = 912).

### SI-based detection of common CNVs

*PennCNV *input SI files for each bull were prepared from an Illumina report containing the SNP name, sample ID, B-allele frequency and log R ratio, using the split option of the kcolumn.pl program in the *PennCNV *package. For a list of the names of these 912 intensity files see list.txt; CNVs were detected using the B-allele frequency file (BovineSNP50K.pfb), the HMM parameter file distributed with the *PennCNV *http://www.openbioinformatics.org/penncnv/penncnv_download.html, and the following command line: perl detect_cnv.pl -test -hmm example.hmm -pfb BovineSNP50K.pfb -conf -log 1.log -out 1.rawcnv -minsnp 1 -lastchr 29 -listfile list.txt. Frequencies for loss and gain of each genetic marker were calculated using a Perl script that counted the total number (N_t_) of copies detected for each marker. Two copies were assumed for each marker that was not included in the *PennCNV *report (1.rawcnv), which contained copy numbers (1, 3, 4, 5) for the CNVs of each bull. Percent frequencies (100|1-Nt/1824|) are reported based on dividing this count by the number of expected chromosomes (1824).

### Real-Time qPCR

Determination of the relative copy number of two amplicons within CNVR #456 (additional file 3) on BTA18 was conducted using a qPCR analysis. The genomic sequence near the extreme SNP positions of this CNVR was analysed for repetitive elements and presence of SNPs http://www.ensembl.org. The primer pairs were designed in repeat and SNP free sequences and were as follows: amplicon I (100 bp) near SNP position 41,760,794 (5'-CTGTTCCTCCAGCATTTCGT-3'; 5'-TTCCTTTTCCCCAGGACTTT-3') and amplicon II (151 bp) near SNP position 41,907,693 (5'-CCATCAGGTTTAAGGGACACA-3'; 5'-CCCCGAAGGTAGAAGTGACA-3'). Gene copy number was normalized to an amplicon of 96 bp of an autosomal reference gene bovine *RPP30 *(GeneID:615098, BTA26, positions 12,893,277-12,893,372) using PCR primers (5'- TGCTTCCATTGTTTCCTGATGA-3'; 5'-TGGGACCAGGTTCCATGATC-3'). RPP30 is used as a reference gene in human CNV studies [22]. No CNV was reported for this gene region in previous studies of CNV in cattle [2,3,13] including this study. Copy number was determined as previously described [23]. Briefly, 5-point standard curve (0.1-62.5 ng of DNA) was generated in duplicate for a mixture of ten reference individuals. Test individuals were assayed in duplicates using 30 ng of DNA per reaction. Absolute Blue SYBER Green ROX Mix (Thermo Fisher scientific, UK) Kit was used for nucleic acid detection. Reactions were performed at 95°C for 15 min followed by 40 cycles of 95°C for 15 s and 60°C for 1 min using an ABI Prism^® ^7000 sequence detection system. Amplification was followed by a dissociation curve analysis to confirm the presence of a single product and the absence of primer dimers. The qbasePLUS software (Biogazelle, Ghent, Belgium) was used for calculation and quality control of relative quantities using *RPP30 *for normalization. Samples that did not pass quality control because of excessive variance between replicates (>0.5 standard error in number of copies per haploid genome) were excluded from further analysis.

### Annotation of CNVs and gene ontology analysis

Gene content of CNVRs was determined using Ensembl http://www.ensembl.org/Bos_taurus/Location/ and Gene Entrez http://www.ncbi.nlm.nih.gov/gene. Genes located up to 250 kb from the CNV borders were regarded as part of the CNVR in cases for which no genes were identified within that region. Since the bovine genome is not well annotated compared to the human genome, we used the human orthologs for gene ontology analyses. The corresponding human GeneIDs were identified using NCBI HomoloGene. When no orthologs were identified using HomoloGene, selection of alternate orthologs was based on BLAST similarity. The PANTHER classification system http://panther6.ai.sri.com/tools/compareToRefListForm.jsp was used to assess the probability of overrepresentation within the list of human orthologs of certain pathways, biological processes and molecular functions using the default Bonferroni correction for multiple testing.

## Authors' contributions

ES conceived the CNV study and its design, performed the bioinformatics and statistical analyses and drafted the manuscript. GG performed the qPCR analysis and participated in the annotation of the CNVs and DNA sample preparation and analysis. AS participated in the DNA sample preparation and analysis. EY participated in the annotation of CNVs. JIW conceived and designed the BeadChip experiment. EE managed the herd book. YZ handled the semen sampling of AI bulls. All authors have read and approved the final manuscript.

## Supplementary Material

Additional file 1 **Detailed mathematical solution for the trinomial expansion of the Hardy-Weinberg principle**. Text in PDF format. Click here for file

Additional file 2 **Allele distribution, χ2 test, r_l _and r_g _values for SNPs of the Illumina BovineSNP50 BeadChip**. Spread sheet in Excel format summarizing the genotypes obtained from the 912 sire samples for all 54,001 genetic markers on BovineSNP50. Click here for file

Additional file 3 **Common CNVs**. Spread sheet in Excel format summarizing the positions and gene content of copy losses (red background) and copy gains (blue background) that displayed significantly high frequency by both the HWE-based and SI-based methods. Previously reported common (frequency > 2.5%) cattle CNVs are also reported using yellow and no colour backgrounds for CNVs confirmed and not confirmed, respectively, by this study. For each CNVR, positions of SNP markers that displayed the same loss or gain status and fell within 0.5 Kb distance from each other are presented in the cell of the position column, and the corresponding estimated frequency is the average frequency of these markers. Click here for file

Additional file 4 **Gene ontology categories in Holstein CNV**. Spread sheet in Excel format. Click here for file
