# Supplementary material for: Analysis of copy loss and gain variations in Holstein cattle autosomes using BeadChip SNPs
Source: BMC Genomics. 2010 Nov 29;11:673. doi: 10.1186/1471-2164-11-673 (PMC3091787; doi:10.1186/1471-2164-11-673)
Supplement: Additional file 1 — Detailed mathematical solution for the trinomial expansion of the Hardy-Weinberg principle. Text in PDF format. [file 1471-2164-11-673-S1.PDF]

Detailed mathematical solution for the trinomial expansion of the Hardy–Weinberg principle: case of a null allele

r_l is frequency of loss

p is frequency of p allele

q is frequency of q allele

Frequency of all possible (3) alleles: $1=r_l+p+q$

Expansion of Hardy-Weinberg: $1=(r_l+p+q)^2$

$1=p^2+2pr_l+q^2+2qr_l+r_l^2+2pq$

#no_calls/#sampled= r_l^2

#Heterozygotes/#sampled=AB=2pq -> pq=AB/2

#p_homozygotes/#sampled=AA=p^2+2pr_l $=p(p+2r_l)$

#q_homozygotes/#sampled=BB=q^2+2qr_l $=q(q+2r_l)$

Multiplying the last two lines:

$AABB=pq(p+2r_l)(q+2r_l)=AB/2(p+2r_l)(q+2r_l)$

$2AABB/AB=(p+2r_l)(q+2r_l)=pq+2pr_l+2qr_l+4r_l^2=pq+2r_l(p+q+r_l+r_l)=AB/2+2r_l(1+r_l)=AB/2+2r_l+2r_l^2$

$0=r_l^2+r_l+AB/4-AABB/AB$

$r_l=\{-1+[1-4(AB/4-AABB/AB)]^{0.5}\}/2=\{[1-AB+4AABB/AB]^{0.5}-1\}/2=[0.25-0.25AB+AABB/AB]^{0.5}-0.5$

Detailed mathematical solution for the trinomial expansion of the Hardy–Weinberg principle: case of an extra (third) allele

r_g is frequency of insertion that produces **pq** chromosome

p is frequency of p allele

q is frequency of q allele

Frequency of all possible (3) alleles: $1=p+q+r_g$

Expansion of Hardy-Weinberg: $1=(p+q+r_g)^2$

$1=p^2+q^2+2pr_g+2qr_g+r_g^2+2pq$

#Heterozygotes/#sampled=$AB=2pr_g+2qr_g+r_g^2+2pq$

#p_homozygotes/#sampled=$AA= p^2$ -> $p=AA^{0.5}$

#q_homozygotes/#sampled=$BB= q^2$ -> $q=BB^{0.5}$

$AB=2AA^{0.5}r_g+2BB^{0.5}r_g+r_g^2+2AA^{0.5}BB^{0.5}$

$0=r_g^2+r_g(2AA^{0.5}+2BB^{0.5})+2AA^{0.5}BB^{0.5}-AB$

$r_g=\{-(2AA^{0.5}+2BB^{0.5})+[(2AA^{0.5}+2BB^{0.5})^2-8AA^{0.5}BB^{0.5}+4AB]^{0.5}\}/2=[AA+2AA^{0.5}BB^{0.5}+BB-2AA^{0.5}BB^{0.5}+AB]^{0.5}-AA^{0.5}-BB^{0.5}=[AA+BB+AB]^{0.5}-AA^{0.5}-BB^{0.5}$
